# ^18^ F-FDG and ^68^ Ga-FAPI PET/CT Imaging in a Patient with High-Grade Meningioma: A Case Report

**DOI:** 10.1055/s-0046-1825774

**Published:** 2026-07-15

**Authors:** Arif Satria Putra Pratama, Hendra Budiawan, Trias Nugrahadi, Achmad Hussein Sundawa Kartamihardja

**Affiliations:** 1 Department of Nuclear Medicine and Molecular Theranostic, Dr. Hasan Sadikin General Hospital/Faculty of Medicine, Universitas Padjadjaran, Bandung, West Java, Indonesia

**Keywords:** ^18^ F-FDG, ^68^ Ga-FAPI, high-grade meningioma, meningioma, molecular imaging, PET/CT

## Abstract

High-grade meningiomas exhibit aggressive biological behavior and may benefit from functional imaging for further characterization. We report a 50-year-old female presenting with a 1-year history of intermittent right-sided headache and recurrent seizures. Contrast-enhanced magnetic resonance imaging demonstrated a well-defined extra-axial mass arising from the right sphenoid wing dura mater, accompanied by hyperostosis, dural tail sign, and vasogenic edema. ^18^ F-FDG and ^68^ Ga-FAPI PET/CT ( ^68^ Ga-fibroblast activation protein inhibitor positron emission tomography/computed tomography) were performed 1 week apart. FDG PET/CT demonstrated a tumor SUVmax of 7.08, comparable to the contralateral substantia nigra (SUVmax 7.27), resulting in limited lesion-to-background contrast (T/N ratio 0.97). In contrast, FAPI PET/CT showed tumor SUVmax of 3.02, with the aorta used as the background reference (SUVmean 1.76), yielding a tumor-to-background ratio of 1.71 and improved visual delineation due to minimal physiological brain uptake. Histopathology confirmed atypical meningioma (World Health Organization grade II). Although FAPI demonstrated higher tumor-to-background contrast than FDG, uptake remained relatively mild, suggesting predominant stromal mesenchymal involvement. Further comparative studies with established modalities such as somatostatin receptor PET are warranted.

## Introduction

Meningioma is the most common primary intracranial tumor in adults. 1 It is generally a slow-growing benign neoplasm from meningothelial (arachnoid) cells. 2 Meningiomas account for 40.8% of all primary brain tumors. Structural imaging modalities such as contrast-enhanced magnetic resonance imaging (MRI) and computed tomography (CT) are routinely used to assess the extent of meningioma lesions. However, these modalities have limitations, particularly in skull-base regions, cases with osseous involvement, and tumors with complex morphology. 3 In suspected residual or recurrent disease, MRI or CT also often cannot reliably distinguish viable tumor tissue from post-radiation changes. 4

Molecular imaging with positron emission tomography (PET) provides complementary functional information beyond structural imaging. 5 6 7 F-2-fluoro-2-deoxy-D-glucose ( ^18^ F-FDG) remains the most widely used tracer for meningioma imaging. Increased FDG uptake is driven by elevated metabolic demand and enhanced glycolysis. A recent meta-analysis reported that FDG PET is helpful in the noninvasive differentiation of benign (World Health Organization [WHO] grade I) meningiomas from atypical (WHO grade II) and anaplastic (WHO grade III) lesions. 8 9 FDG PET also has demonstrated utility in identifying metastatic malignant meningioma presenting as extracranial high-grade soft tissue lesions. 10 However, its diagnostic performance for intracranial lesions is often limited by high physiological uptake in the normal cerebral tissue, which reduces lesion-to-background contrast.

In addition to FDG, somatostatin receptor (SSTR) PET imaging has emerged as a highly sensitive modality for meningioma evaluation due to overexpression of SSTR2 in tumor cells. 11 Radiotracers such as ^68^ Ga-DOTATATE and ^68^ Ga-DOTATOC have demonstrated excellent lesion detection capability. A recent systematic review demonstrated that SSTR PET provides consistently high diagnostic accuracy in meningioma, outperforming MRI, particularly in skull-base, small-volume, and recurrent disease, and significantly influencing radiotherapy planning and overall clinical management. These findings establish SSTR PET as a reference functional imaging modality in meningioma assessment. 12

Fibroblast activation protein (FAP), in contrast, represents a different biological target. FAP is a serine protease expressed within the tumor-associated stromal microenvironment and has emerged as an important oncologic imaging target. 13 Radiopharmaceuticals labeled with FAP inhibitors (FAPIs), such as ^68^ Ga-FAPI, demonstrate strong uptake in tumors with high FAP expression. A study evaluating FAP expression in meningioma demonstrated high expression in WHO grade II and III lesions. 14 Unlike FDG, FAPI tracers show minimal physiological uptake in normal brain tissue, potentially enabling improved tumor delineation. However, the clinical role of FAPI PET/CT in meningioma remains incompletely defined.

This case report presents a comparative assessment of ^18^ F-FDG and ^68^ Ga-FAPI PET/CT imaging in a patient with high-grade meningioma.

## Case Presentation

A 52-year-old female presented with a 1-week history of worsening headache accompanied by recurrent focal seizures. The patient had experienced intermittent seizure episodes over the previous year but had not undergone advanced neuroimaging evaluation. Prior to referral for PET/CT imaging, she received symptomatic and supportive therapy consisting of intravenous 0.9% NaCl (1500 mL/24 hours), paracetamol (500 mg three times daily), codeine (30 mg three times daily), levetiracetam (500 mg twice daily), and a dexamethasone loading dose of 10 mg intravenously followed by 5 mg four times daily.

**Fig. 1:**
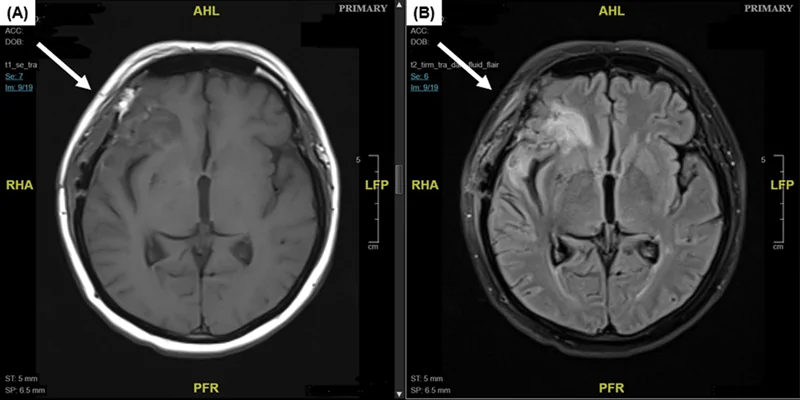
Magnetic resonance imaging (MRI) of the brain demonstrating an extra-axial mass arising from the right sphenoid wing. ( **A** ) T1-weighted image showing a hypointense extra-axial lesion (white arrow). ( **B** ) T2-FLAIR image demonstrating the lesion with surrounding vasogenic edema (white arrow).

Contrast-enhanced MRI demonstrated a well-defined extra-axial mass arising from the right sphenoid wing dura mater, associated with adjacent hyperostosis. The lesion exhibited regular margins and a positive dural tail sign. It caused compression of the right lateral ventricle and was surrounded by vasogenic edema. On MRI sequences, the mass appeared hypointense on T1-weighted imaging and isointense on T2-weighted imaging and T2-FLAIR sequences. No restricted diffusion was observed on diffusion-weighted imaging with corresponding apparent diffusion coefficient mapping, and no blooming artifact was detected on susceptibility-weighted imaging. Post-contrast imaging demonstrated strong homogeneous enhancement, raising suspicion for dural metastasis versus high-grade meningioma ( Fig. 1 ).

**Fig. 2:**
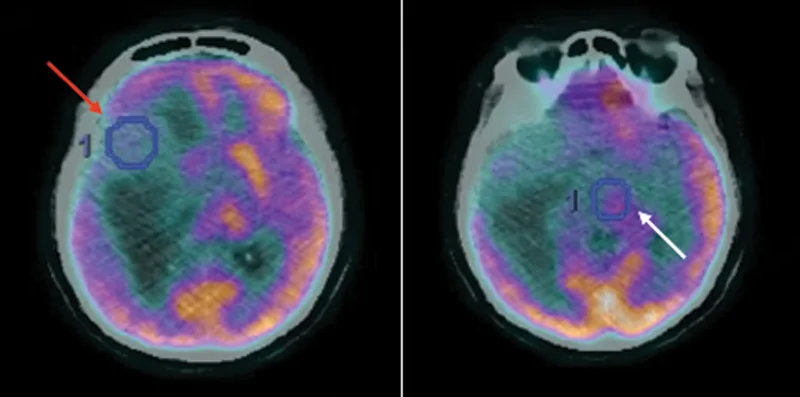
Left: ^18^ F-FDG PET/CT imaging of the brain demonstrating FDG uptake in the right sphenoid wing lesion (red arrow) with activity comparable to surrounding brain tissue. Right: the contralateral substantia nigra was used as the background reference (white arrow). Both tumor and background ROIs are indicated by blue circles. CT, computed tomography; PET, positron imaging tomography; ROI, region of interest.

Subsequently, the patient underwent ^18^ F-FDG PET/CT for metabolic evaluation. The study demonstrated increased tracer uptake in the right sphenoid wing lesion corresponding to the enhancing component seen on MRI ( Fig. 2 ). Tumor uptake was quantified using SUVmax, measuring 7.08. Background activity for tumor-to-background ratio calculation was measured using SUVmax obtained from a region of interest placed over the contralateral substantia nigra, measuring 7.27. The resulting tumor-to-background ratio was 0.97, indicating limited lesion conspicuity due to high physiological cerebral glucose metabolism.

**Fig. 3:**
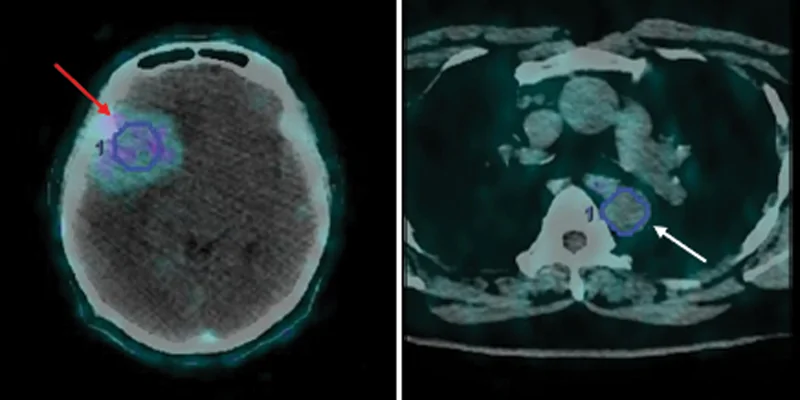
Left: ^68^ Ga-FAPI PET/CT imaging demonstrating mild tracer uptake in the right sphenoid wing lesion (red arrow). Right: the descending thoracic aorta was used as the background reference (white arrow). Both tumor and background ROIs are indicated by blue circles. CT, computed tomography; PET, positron imaging tomography; ROI, region of interest.

One week later, ^68^ Ga-FAPI PET/CT was performed ( Fig. 3 ). Mild radiotracer uptake was observed in the same lesion, with a tumor SUVmax of 3.02. Background activity was assessed using SUVmean obtained from a region of interest placed within the lumen of the descending thoracic aorta, measuring 1.76. This yielded a tumor-to-background ratio of 1.71, demonstrating improved visual contrast compared with FDG despite lower absolute tumor uptake.

Histopathological examination following surgical resection confirmed atypical meningioma (WHO grade II).

## Discussion

FDG PET has been investigated as a noninvasive tool for assessing meningioma grade according to the WHO classification. Several studies have reported higher FDG-derived parameters—such as SUV and tumor-to-normal tissue (T/N) ratio—in atypical and anaplastic meningiomas compared with grade I lesions. 8 15 16 A prior study suggested that a T/N ratio ≥1.0, using the substantia nigra as reference tissue, may indicate high-grade meningioma with high specificity. 9 However, findings across studies remain inconsistent, and the diagnostic performance of FDG PET in individual cases is variable. 17 18

In the present case, tumor FDG uptake was comparable to that of the contralateral substantia nigra, yielding a T/N ratio below 1. Although histopathology confirmed atypical meningioma (WHO grade II), the lesion did not demonstrate markedly elevated FDG uptake relative to background brain structures. This finding highlights a known limitation of FDG in intracranial tumor imaging: high physiological cerebral glucose metabolism can reduce lesion conspicuity and compromise tumor delineation. 7 Moreover, meningiomas are generally slow-growing tumors that may exhibit only moderate increases in glucose metabolism, further limiting contrast resolution. 18

In contrast, ^68^ Ga-FAPI PET/CT demonstrated visually clearer tumor delineation with a higher tumor-to-background ratio, despite lower absolute SUV compared with FDG. The improved contrast primarily reflects minimal physiological FAPI uptake in normal brain tissue. 19 Unlike FDG, FAPI tracers do not cross an intact blood–brain barrier (BBB). Therefore, intracranial tracer accumulation suggests BBB disruption or stromal activation within the tumor microenvironment. 20 21

Emerging evidence indicates that in brain tumors, FAPI uptake predominantly reflects tumor-associated stromal mesenchymal cells, including perivascular mesenchymal cells and myofibroblast-like cells, rather than classical cancer-associated fibroblasts. 19 This distinction is particularly relevant in meningioma, which arises from meningothelial cells and exhibits mesenchymal characteristics. 22 Prior studies have demonstrated FAP expression in higher grade meningiomas. 14 Nevertheless, FAPI uptake intensity varies, and in the present case, tracer accumulation remained relatively mild, suggesting limited stromal FAP expression despite improved visual contrast.

When interpreted alongside established functional imaging modalities, particularly SSTR PET, which directly targets SSTR2 expression on tumor cells, FAPI PET appears to represent a different biological compartment. SSTR PET has demonstrated consistently high diagnostic accuracy and strong tracer uptake in meningioma, and remains the reference molecular imaging modality in this disease entity. 23 In comparison, FAPI PET may provide complementary information reflecting stromal biology rather than tumor receptor density. 24

## Conclusion

In this case of atypical meningioma, ^68^ Ga-FAPI PET/CT provided improved lesion conspicuity compared with ^18^ F-FDG PET/CT due to lower physiological brain background activity, although tracer uptake intensity remained modest. These findings suggest that FAPI imaging reflects a tumor characteristic that differs from glucose metabolism and should be interpreted cautiously in the context of histological grading.

While SSTR–based PET remains the established molecular imaging standard in meningioma, FAPI PET may offer complementary insights and warrants further investigation to clarify its potential clinical role.
